# The role of neoantigen in immune checkpoint blockade therapy

**DOI:** 10.1186/s40164-018-0120-y

**Published:** 2018-11-16

**Authors:** Ming Yi, Shuang Qin, Weiheng Zhao, Shengnan Yu, Qian Chu, Kongming Wu

**Affiliations:** Department of Oncology, Tongji Hospital of Tongji Medical College, Huazhong University of Science and Technology, Wuhan, 430030 China

**Keywords:** Neoantigen, PD-1/PD-L1, CTLA-4, Immunotherapy, Cancer vaccine, Adoptive T cell transfer

## Abstract

Immune checkpoint inhibitor induces tumor rejection by activated host immune system. The anti-tumor immune response consists of capture, presentation, recognition of neoantigen, as well as subsequent killing of tumor cell. Due to the interdependence among this series of stepwise events, neoantigen profoundly influences the efficacy of anti-immune checkpoint therapy. Moreover, the neoantigen-specific T cell reactivity is the cornerstone of multiple immunotherapies. In fact, several strategies targeting neoantigen have been attempted for synergetic effect with immune checkpoint inhibitor. Increasing neoantigen presentation to immune system by oncolytic virus, radiotherapy, or cancer vaccine is feasible to enhance neoantigen-specific T cell reactivity in theory. However, some obstacles have not been overcome in practice such as dynamic variation of neoantigen landscape, identification of potential neoantigen, maintenance of high T cell titer post vaccination. In addition, adoptive T cell transfer is another approach to enhance neoantigen-specific T cell reactivity, especially for patients with severe immunosuppression. In this review, we highlighted the advancements of neoantigen and innovative explorations of utilization of neoantigen repertoire in immune checkpoint blockade therapy.

## Introduction

**Fig. 1:**
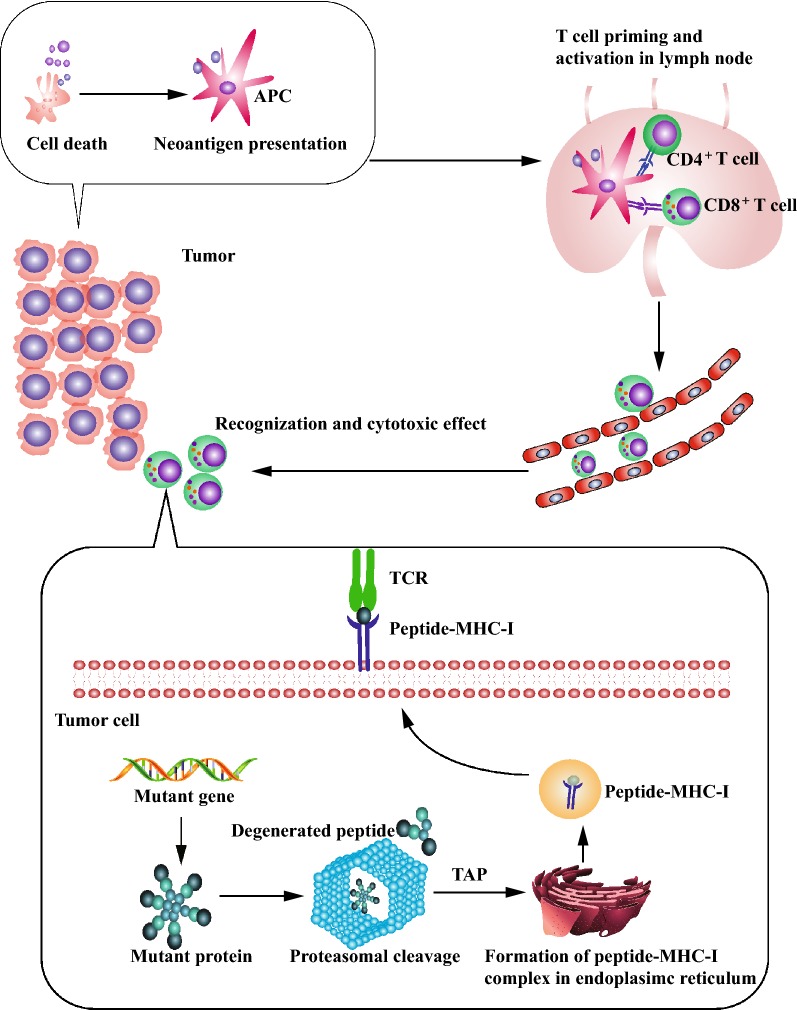
Cancer-Immunity cycle and neoantigen presentation. cancer-immunity cycle: neoantigen released by dead cancer cell initiates the anti-tumor immune response. Then the neoantigen is captured and presented by antigen presentation cell (APC) which induces the priming and activation of neoantigen-specific T cell in peripheral immune organ. Peripheral activated T effector cell traffics to and infiltrates into tumor bed. Following recognition of neoantigen, tumor cell is killed by tumor infiltrating lymphocyte (TIL). Neoantigen presentation: in the proteasome of tumor cell, mutant protein derived from somatic mutation is degenerated into peptide and then transported to endoplasmic reticulum. The peptide binds to major histocompatibility complex I (MHC-I) binding site by transporter associated with antigen processing (TAP). Simultaneously, the assembled peptide-MHC-I complex is transported to membrane of tumor cell. Cytotoxic T cell could recognize peptide-MHC-I complex and kill the tumor cell

It is well established that non-self-antigen generated by tumor somatic mutation confers tumor immunogenicity and induces anti-tumor immune response [1]. Cancer immunotherapies such as chimeric antigen receptor T cell, bispecific antibody, cancer vaccine, and immune checkpoint inhibitor, eradicate tumor cell by enhancing host cancer-specific immune reactivity [2–5]. In 2013, Cancer-Immunity cycle theory was established to describe anti-tumor immune response (Fig. 1) [6]. In the cycle, mutation-derived neoantigen is released by cancer cell and initiates the anti-tumor immune response. Then the neoantigen is captured and presented by professional antigen presentation cell (APC) which induces the priming and activation of neoantigen-specific T cell in peripheral immune organ. Peripheral activated T effector cell traffics to and infiltrates into tumor bed. Following recognition of neoantigen, tumor cell is killed by tumor infiltrating lymphocyte (TIL). During cancer immune evasion, one or more steps are undermined [6].

Strategies such as enhancing release and presentation of neoantigen, increasing neoantigen-specific T cell abundance, or blocking immune checkpoint, impact different steps in Cancer-Immunity cycle. Among these strategies, immune checkpoint blockade attracts intensive attention for potent and durable tumor control [7]. Cytotoxic T lymphocyte-associated antigen-4 (CTLA-4) antibody primarily blocks inhibitory signaling of T cell priming and activation while programmed cell death protein 1/programmed cell death ligand 1 (PD-1/PD-L1) antibody primarily recovers attenuated anti-tumor immune response in tumor bed [6]. However, due to the interdependence of multiple steps of cancer-immunity cycle, the efficacy of immune checkpoint blockade treatment is substantially affected by neoantigen presentation and recognition [6]. Therefore, it is presumed that neoantigen is a predictive biomarker and synergistic treatment target for immune checkpoint inhibitor.

## Predictive value of neoantigen in immune checkpoint blockade therapy

### Tumor somatic mutation

Somatic mutation participates in tumor initiation and progression. In the meanwhile, tumor mutation landscape influences immune surveillance and evasion. In the proteasome of tumor cell, mutant protein derived from somatic mutation is degenerated into peptide and then transported to endoplasmic reticulum. The peptide binds to major histocompatibility complex I (MHC-I) binding site. Simultaneously, the assembled peptide-MHC-I complex is transported to membrane of tumor cell (Fig. 1). Cytotoxic T cell could recognize peptide-MHC-I complex and kill the tumor cell [8].

**Fig. 2:**
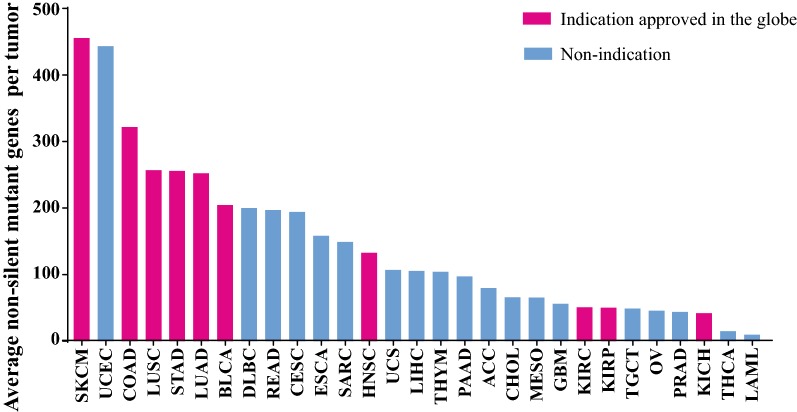
The number of mutant gene across 29 types of tumor. Average mutant gene number is calculated based on TCGA datasets (https://tcga.xenahubs.net). *ACC* adrenocortical cancer, *BLCA* bladder cancer, *CESC* cervical cancer, *CHOL* cholangiocarcinoma, *COAD* colon adenocarcinoma, *DLBC* lymphoid neoplasm diffuse large B-cell lymphoma, *ESCA* esophageal carcinoma, *GBM* glioblastoma multiforme, *HNSC* head & neck squamous cell carcinoma, *KICH* kidney chromophobe cancer, *KIRC* Kidney renal clear cell carcinoma, *KIRP* kidney papillary cell carcinoma, *LAML* acute myeloid leukemia, *LIHC* hepatocellular carcinoma, *LUAD* lung adenocarcinoma, *LUSC* lung squamous cell carcinoma, *MESO* mesothelioma, *OV* ovarian serous cystadenocarcinoma, *PAAD* pancreatic adenocarcinoma, *PRAD* prostate adenocarcinoma, *READ* rectal cancer, *SARC* sarcoma, *SKCM* melanoma, *STAD* stomach adenocarcinoma, *TGCT* testicular germ cell tumor, *THCA* thyroid cancer, *THYM* thymoma, *UCEC* endometrioid cancer, *UCS* uterine carcinosarcoma

Neoantigen derives from tumor somatic mutation, thus tumor mutation burden (TMB) is considered as the surrogate of neoantigen burden and the predictive biomarker of checkpoint blockade therapy [9–11]. Effective tumor regression induced by immune checkpoint blockade therapy is commonly observed in some specific cancer types [12]. A main factor contributing to different treatment effect among various type of cancers is TMB [13]. Based on data from TCGA, tumor mutation burden analysis across multiple cancers was conducted (Fig. 2). The result showed that cancer types which are approved for immune checkpoint inhibitor therapy such as melanoma, bladder cancer, and head and neck cancer tend to harbor high TMB. As an exception, three types of renal cell cancer have relative low TMB, but they response to nivolumab well. The sensitivity of kidney cell cancer to PD-L1 antibody is attributed to high-frequency indel variation-derived frameshift mutation. It is found that the probability of frameshift mutation generating neoantigen is eight times higher than non-synonymous single nucleotide variation [13].

The treatment effect not only varies with tumor type, but also positively relates to TMB in patients with the same type of tumor [14, 15]. Rizvi et al. investigated relationship between efficacy of PD-1 blockade and TMB in non-small cell lung cancer (NSCLC). The result showed that patients with high TMB showed a significant advantage in progression-free survival (PFS) than low TMB group (Hazard Ratio = 0.19, log-rank *P* = 0.0004) [14]. Subsequently, Carbone et al. further confirmed the correlation between TMB and efficacy of nivolumab in stage IV or recurrent NSCLC patients [16].

For some tumors, especially gastrointestinal tumor, accumulated somatic mutations closely relate to mismatch repair deficiency (dMMR) [5]. Mismatch repair system participates in correcting base substitution, insertion, and deletion in the process of DNA replication [17]. It is generally thought that high microsatellite instability (MSI-H)/dMMR heralds the clinic benefit from immune checkpoint inhibitors [15, 18–20]. Le et al. evaluated the relationship between dMMR and efficacy of PD-1 inhibitor across 12 tumor types. Objective radiographic response rate was up to 53% in patients with dMMR, indirectly indicating the predictive role of TMB in immune checkpoint blockade treatment [15].

### Predictive value of neoantigen in immune checkpoint inhibitor

Though high TMB and neoantigen burden contribute to high response rate to immune checkpoint inhibitor generally, the two biomarkers could not fully determinate treatment effect. For example, in NSCLC patients undergoing PD-1 or PD-1/CTLA-4 inhibitor treatment, it was detected that both TMB and candidate neoantigen level of post-progression tumor tissue were higher compared to pre-immunotherapy tumor tissue [21]. By analysis of landscape of tumor somatic mutation, it was observed that some mutant genes encoding neoantigens were eliminated while some mutations not encoding neoantigen were gained after resistance to anti-immune checkpoint therapy [21]. Besides, the eliminated neoantigen was found to have higher MHC binding affinity compared with retained and gained neoantigen [21]. Further exploration revealed that eliminated mutations harboring altered domain related MHC binding and T cell receptor (TCR) binding [21]. By autologous T cell culture, the eliminated neoantigen could induce clonal T cell expansion successfully, indicating that the loss of these antigen might relate to immune escape and resistance to immunotherapy [21].

It is notable that as a widely adopted biomarker for patient selection and efficacy prediction prior to immune checkpoint blockade therapy, TMB is not a perfect surrogate of immunogenic neoantigen. Actually, anti-tumor immune response is initiated by the recognition of neoantigen-MHC complex rather than mutated gene [22]. In the “lottery of neoantigen formation”, the production of neoantigen related with clinic benefit is a probabilistic event. High TMB could elevate the probability of production of immunogenic neoantigen but could not guarantee the occurrence of neoantigen-specific response [1, 23]. Snyder et al. noticed that some melanoma patients failed to response to CTLA-4 inhibitor even harboring high TMB. Further investigation showed that patients benefiting from CTLA-4 inhibitor tented to have neoantigens containing the same tetrapeptide, which was absent in the patients resistant to CTLA-4 inhibitor [23]. Moreover, therapeutic benefit-related neoantigens were found to resemble epitopes from pathogen which had strong immunogenicity [23]. Intriguingly, the homology between tumor neoantigen and pathogen epitope was speculated to relate with cross-reactivity of immunity [23]. Some observations showed that antigen from microbiota influenced the efficacy of immune checkpoint inhibitor [24–27].

## Prediction of neoantigen

**Fig. 3:**
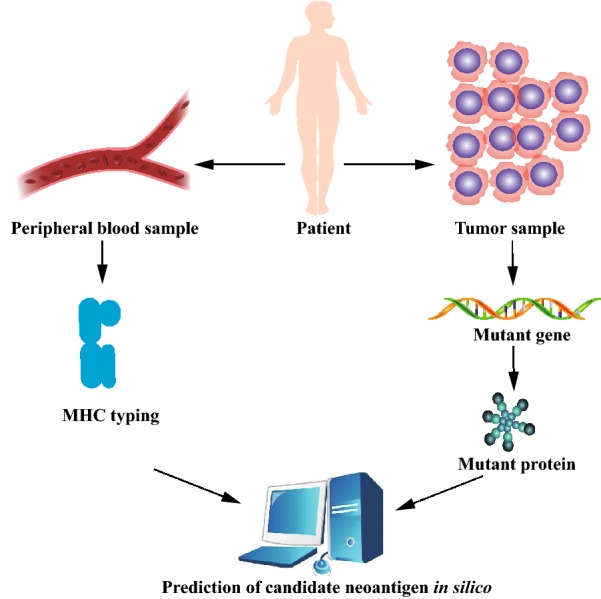
Prediction of candidate neoantigen. The common prediction algorithm consists of three parts: **a** identification of mutated protein; **b** identification of major histocompatibility complex (MHC) typing; **c** prediction of the binding affinity between MHC and neo-peptides

For further application of neoantigen as a predictive biomarker or a therapeutic target for checkpoint blockade therapy, the prediction of candidate neoantigen is indispensable. The common prediction algorithm consists of three parts: (A) identification of mutated protein; (B) identification of MHC typing; (C) prediction of the binding affinity between MHC and neo-peptides (Fig. 3) [28, 29].

Next generation sequencing (NGS) provides an available access to obtain somatic mutation data in the whole genome. Based on public databases of gene, transcription, and protein sequence, annotation software interprets the data from NGS to expression status of protein [30, 31]. In terms of identification of MHC typing, as the most polymorphic region, MHC typing is usually obtained by sequencing data which could be further interpreted by well-annotated sequences in public databases [32–35].

In the process of antigen recognition, only 0.5% peptides could bind to MHC, which is the most selective step [36]. Therefore, MHC binding affinity is the core parameter for most prediction algorithms of candidate neoantigen [37]. In most cases, the researchers tend to focus on binding affinity of MHC-I molecule. The primary reason is that MHC-I molecule directly relates with neoantigen presentation on tumor cell [38, 39]. Besides, peptides binding to MHC-I molecules are usually distributed in narrow length, mainly in 8 and 11 amino acids while MHC-II molecules could bind to peptides which are distributed in broader length (from 11 to 30 amino acids) [28, 40]. It is more available to utilize MHC-I molecule for neoantigen prediction in silico. Valuation of affinity is mainly conducted by two methods: 3D structure-based prediction or sequence-based method [28]. Propelled by NGS, sequence-based methods have been developing, from previously verified peptide binders of a specific MHC allele to nonlinear methods [41]. However, due to the limited amount of detected MHC allele, machine learning-based method was created to overcome the problem. Beyond MHC allele have been detected, trained neuron network could calculate the affinity of given peptide-MHC complex by mimicking MHC sequence from peptide-binding residues, called pan-specific tool [42].

Though multiple tools have been created (Table 1), high false positive rate of neoantigen prediction could not be ignored. Some steps of neoantigen processing prior to MHC binding including cleavage in proteasome and transportation to endoplasmic reticulum influence the effect of neoantigen presentation [43]. Therefore, comprehensive algorithm involving affinity of peptide-MHC complex, cleavage in proteasome, and transporter associated with antigen processing (TAP) shows better performance than analysis with single approach [44, 45]. Some prediction algorithms such as NetTepi even contain parameters including binding stability and T cell propensity [46].

**Table 1:** Algorithm for neoantigen prediction

Algorithm	Brief description of algorithm	Refs.
NetMHC-3.0	Artificial neural network-based algorithm for prediction of binding affinity between MHC-I and peptides of length 8–11	[89]
NetMHCpan-3.0	Machine-learning model-based algorithm for prediction of binding affinity between MHC-I and peptides, a pan-specific version	[90]
NetMHCcons	Comprehensive algorithm for prediction of binding affinity between MHC-I and peptides	[41]
NetMHCstab	Artificial neural network-based algorithm for prediction of stability of peptide-MHC-I complex	[91]
PickPocket	Position-specific scoring matrix-based algorithm for prediction of binding affinity between MHC-I and peptides	[92]
FRED2	Epitope prediction for neoantigen	[93]
NetCTL-1.2	Comprehensive prediction algorithm containing proteasome cleavage, TAP transport, and MHC-I binding affinity	[94]
NetCTLpan	The pan-specific version of NetCTL	[95]
NetTepi	Integrated prediction algorithm containing binding affinity, stability of peptide-MHC-I complex, and T cell propensity	[46]
pVAC-Seq	Identification of neoantigen by tumor mutation and expression data	[96]
EpiToolKit	Prediction of MHC-I typing and T cell epitope	[97]
WAPP	Comprehensive prediction algorithm containing proteasome cleavage, TAP transport, and MHC-I binding affinity	[98]

*MHC* major histocompatibility complex, *TAP* transporter associated with antigen processing

Due to decreased false positive rate benefiting from improved algorithm, it is more available to analyze the shared features among the candidate neoantigens for potential therapeutic target [47]. Kim et al. designed a novel algorithm consisting of peptide-MHC affinity and other immunogenicity-related parameters for candidate neoantigen prediction [36]. Notably, just 16 out of 3760 candidate neoantigens were detected in more than one sample. Besides, analysis of 1867 known neoantigens revealed that most neoantigens were homological with pathogens such as Mycobacterium tuberculosis, Trypanosoma cruzi, Vaccinia virus, Human herpes virus, and Hepatitis C virus [36].

## Therapeutic application of neoantigen in immune checkpoint blockade therapy

### Increased release of neoantigen to immune system

Restricted antigen presentation renders some neoantigens hidden from immune surveillance [48]. However, these neoantigens could be released post cancer cell death. Released neoantigen has a probability to induce the emergency of new T cell clones which could further circulate and kill other cancer cells [48]. Interventions such as oncolytic virus and radiotherapy effectively eliminate local cancer cells that might influence the immune surveillance status systemically [48]. Combination therapy of these interventions and immune checkpoint blockade showed more potent tumor eradication than mono-therapy of immune checkpoint inhibitor [1].

#### Immune checkpoint inhibitor combined with oncolytic virus

Distinguished from normal cell, tumor cell tends to have an undermined capability to counteract virus infection [49]. Therefore, oncolytic virus preferentially infects cancer cell and induces lysis of cancer cell subsequently. By deleting or silencing gene which is essential to virus replication in normal cell, engineered oncolytic virus is designed for higher tumor specificity [50]. In addition to direct cancer cell lysis effect, oncolytic virus induces local and systemic tumor specific immune response which might influence tumor surveillance [51, 52]. After infection by oncolytic virus, cancer cell upregulates the production of reactive oxygen species and some cytokines (e.g. interleukin-2 and interferon-γ) to counteract the infection [48]. Subsequently activated innate immune response and magnified adaptive immune response promote recognition of tumor associated antigen and neoantigen [48]. Characteristics of oncolytic virus including tumor cell killing and immune stimulatory effect are favorable to immune checkpoint blockade therapy [53–55].

Woller et al. found the synthetic effect of oncolytic virus with immune checkpoint inhibitor therapy [52]. In mice model bearing disseminated CMT64 tumor, researchers observed that neither PD-1 inhibitor nor oncolytic virus alone could inhibit tumor progression [52]. However, the combination therapy effectively inhibit the growth of primary and metastatic lesions [52]. Systemic resistance to PD-1 inhibitor was overcome by local oncolytic virus infection, which was primarily attributed to broadened neoantigen spectrum and elevated inflammation magnitude [52]. Similarly, Zamarin et al. observed that the intratumoral oncolytic virus treatment combined with PD-1 inhibitor resulted in regression of primary and distant tumor lesions, suggesting the potential application of oncolytic virus for overcoming immune checkpoint inhibitor resistance [56].

#### Immune checkpoint inhibitor combined with radiotherapy

Radiotherapy substantially influences tumor immunogenicity and tumor microenvironment [57, 58]. Actually, radiotherapy-induced tumor cell death releases neoantigen which is equal to a tumor vaccine in situ [59]. Concurrent neoantigen specific immune response is activated accompanied with abundant T cell infiltration [60]. The conversion of tumor immune microenvironment from “cold” to “hot” synergizes with immune checkpoint inhibitors, enhancing anti-tumor response by different steps of Cancer-Immunity cycle [61, 62].

Aboudaram et al. interrogated the treatment effect of concurrent radiotherapy and PD-1 inhibitor (pembrolizumab or nivolumab) [63]. It was showed that metastatic melanoma patients receiving PD-1 inhibitor and concurrent radiotherapy had higher objective response rate than patients receiving PD-1 inhibitor monotherapy (64.7% vs. 33.3%, *P *= 0.02) [63]. In addition, in the NSCLC patients, Fiorica et al. observed the similar phenomenon [64]. The results suggested that nivolumab combined concurrent radiotherapy had advantage over nivolumab monotherapy in prognosis (1-year overall survival rate: 57.8% vs 27.4%, *P *= 0.043; 1-year progression-free survival rate: 57.8% vs 20.6%, *P *= 0.040) [64].

Apart from increased release of neoantigen, radiotherapy bi-directionally influences the infiltration of immune cells. On the one hand, radiotherapy alters the expression of adhesion molecules on tumor vasculature endothelial cells (e.g. upregulated selectins vascular cell adhesion molecule-1, and intercellular adhesion molecules 1), which are helpful to recruitment and infiltration of immune cell [65]. On the other hand, radiotherapy-induced cytokines including transforming growth factor-β (TGF-β) participate in remodeling of extracellular matrix. TGF-β could promote the production of extracellular matrix protein which impedes the infiltration of immune cell into tumor bed [65]. Notably, the inhibitory tumor immune microenvironment could be counteracted by ICI treatment. It was observed that the α-CTLA-4 treatment modulated cytokine milieu including the upregulated interleukin-2 and downregulated TGF-β, indicating the synthetic effect of ICI treatment and radiotherapy [65].

### Neoantigen-based cancer vaccine

Compared with defined molecular neoantigen vaccine, neoantigen release induced by interventions such as oncolytic virus and radiotherapy is unpredictable. Neoantigen released by tumor cell death is diluted by larger amount of non-mutant peptides, decreasing the probability of neoantigen presentation and recognition [1]. Thus, neoantigen vaccine containing candidate neoantigen would be a more effective synthetic therapy for immune checkpoint inhibitor [66]. As a monotherapy, neoantigen vaccine is not sufficient for tumor control primarily due to inhibitory tumor immune microenvironment. However, the combination therapy with immune checkpoint inhibitor overcomes the obstacle and shows promising application prospect [47, 67].

#### Neoantigen cancer vaccine-induced tumor rejection

In the immune response induced by neoantigen vaccine, neoantigen is mainly recognized by cross-presentation [47]. Following professional APC recruited to the vaccination site, activated APC migrates to drain lymph node and induces the activation of T cell [47]. For the design of neoantigen vaccine, selection of neoantigen which could be effectively presented and recognized is a technical challenge. In addition to immune-stimulating components, different adjuvants and vaccine platforms influence efficacy of vaccine as well [66, 68, 69]. Actually, platforms including tumor cell, DNA, RNA, viral, bacteria, dendritic cell, and peptide/protein have been applied in vaccine design [66]. Among vaccine platforms mentioned above, viral based platform vaccine shows the potentiality to induce potent and durable T cell response [47]. Moreover, tumor could not be eliminated completely in a short time, so long term tumor control needs both prime vaccination and multiple boost vaccinations to maintain T cell activity.

Based on mice model bearing MCA-induced sarcomas, Gubin et al. conducted a trial to explore the effect of neoantigen vaccination on tumor control [70]. By comprehensive analysis containing Stabilized Matrix Method algorithm, the Artificial Neural Network algorithm, and the NetMHCpan algorithm, the affinity and stability of peptide-MHC I complex were calculated among all non-synonymous mutations [70]. Subsequently, candidate neoantigens were filtered by the following standards: A. screening out the neoantigen poorly processed in proteasome; B. eliminating neoantigens with lower binding affinity to MHC I than their corresponding wild type (WT) peptides. Finally, two predominant H-2 K^b^ restricted candidate epitopes were identified: an A506T mutation in Asparagine-linked glycosylation 8 (mAlg8) and a G1254V mutation in Laminin alpha subunit 4 (mLama4) [70]. To verify the role of mAlg8 and mLama4 in anti-PD-1 treatment, in the mice model experiencing anti-PD-1-induced sarcomas (d42m1-T3) rejection, researchers isolated CD8^+^ T cell from spleen. Intriguingly, the isolated T cell could be stimulated to produce interferon-γ by mAlg8 and mLama4 rather than other antigens [70]. Moreover, selected reaction monitoring showed that mLama4 and mAlg8 were the only candidate epitopes with strong binding affinity in the H-2K^b^ eluate [70]. The observation that mAlg8 or mLama4 specific CD8^+^ T cell increased temporally after anti-PD-1 treatment and mounted to peak value just before tumor regression further indicated the role of these neo-epitopes [70]. Given the results mentioned above, researchers designed a cancer vaccine mainly consisting of mAlg8 or mLama4. The vaccine led to potent tumor elimination in mice model compared with the control mice [70].

#### Immune checkpoint inhibitor combined with neoantigen cancer vaccine

On the one hand, some failures to response to neoantigen vaccines are primarily attributed to suppressive tumor microenvironment. Emerging immune modulators including anti-PD-1/PD-L1 antibody, anti-CTLA-4 antibody, and anti-T cell immunoglobulin and mucin domain-containing protein-3 (Tim-3) antibody could resolve the problem [71, 72]. Sahin et al. conducted a study to explore the treatment effect of RNA platform-based neoantigen cancer vaccine [73]. One out of three melanoma patients receiving vaccine experienced relapse and distant metastasis. However, by subsequent pembrolizumab treatment, the patient showed a complete response [73]. Compared with the reported complete response rate (below 10%), this treatment effect is satisfactory [73]. Further investigation revealed that neoantigen specific T cell was PD-1^+^, and the expression abundance of PD-L1 in tumor tissue was upregulated, suggesting the suppressive immune microenvironment induced by neoantigen cancer vaccine [73]. Presumably due to blockaded inhibitory immune regulation, the combination therapy showed more robust tumor control effect [73]. Simultaneously, Ott et al. investigated the efficacy of neoantigen cancer vaccine targeting up to 20 predicted neo-epitopes [74]. It was showed that 2 out of 6 melanoma patients experienced tumor relapse [74]. Similarly to the phenomenon mentioned above, both 2 recurrent melanoma patients had a complete tumor rejection after pembrolizumab treatment, which further verified the feasibility of combination therapy [74].

On the other hand, frequently-occurring adaptive resistance during immune checkpoint inhibitor is related with variation of neoantigen repertoire [67]. Due to heterogeneity of tumor, part of mutations are shared by all tumor cells while the others are exclusively expressed by subpopulations [75]. Under survival selective pressure, subpopulations sensitive to immune checkpoint inhibitor are eliminated. In the meanwhile, subpopulations resistant to immune checkpoint inhibitor have an advantage in proliferation [76]. As a result, loss of immunologic epitopes results in alternative subpopulation constitution and resistance to treatment, called immunoediting [77, 78]. However, the resistance could be overcome by neoantigen cancer vaccine, because immune-stimulating component of vaccine could be manipulated depending on dynamic variation of neoantigen spectrum during tumor evolution [67]. Carreno et al. conducted a study to investigate the influence of neoantigen cancer vaccine on neoantigen-specific T cell receptor repertoire [79]. The study recruited 3 melanoma patients which had been treated with ipilimumab [79]. Each patient received DC platform-based neoantigen cancer vaccine which containing 7 identified neoantigens [79]. Before and after vaccination, researchers collected peripheral blood sample and estimated the immune response to supposed neoantigens [79]. Immune monitoring showed that T cell response targeting these neoantigens was enhanced. Moreover, compared with pre-vaccination, vaccination induced T cell response to 2 additional neoantigens per patient [79]. Subsequently, composition and abundance of neoantigen-specific T cell was analyzed. In the purified CD8^+^ T cell isolated from peripheral blood, researchers found that after vaccination, the frequency of existing neoantigen-specific TCRβ clonotypes were increased accompanied with additional clonotypes for all each neoantigen [79]. The results showed that both TCRβ clonotypes targeting predominant and sub-predominant neoantigens were elevated after vaccination, suggesting the broadened spectrum of T cell response [79]. Two patients recruited in the study were resistant to ipilimumab and had recurrent tumor lesions. By intervention of neoantigen cancer vaccine, effective anti-tumor immune response was rebuilt [79].

### Adoptive T cell transfer

The efficacy of immune checkpoint inhibitor directly depends on activity of neoantigen specific T cell [80]. Therefore, manipulating composition and abundance of T cell would be another approach to enhance treatment effect of immune checkpoint inhibitor [80]. In preclinical trials, adoptive T cell transfer targeting tumor specific mutations showed potent anti-tumor activity [81, 82]. In 2014, Tran et al. conducted a study to explore the treatment effect of adoptive T cell transfer in a patient with metastatic cholangiocarcinoma [83]. After identification of specific T cell clone targeting tumor specific mutation (mutation of erbb2 interacting protein, called ERBB2IP), autologous TIL was stimulated by interleukin-2 for proliferation and enhanced activity [83]. Subsequently, total 42.4 billion TILs were transferred to the patient which contained nearly 10 billion ERBB2IP mutation specific CD4^+^ T cells [83]. Prior to adoptive T cell transfer, the patient had received multiple chemotherapy regimens and tumor had metastasized to liver and lung [83]. Following the T cell injection, all lesions in liver and lung showed regression and reached a maximum reduction up to 30% [83]. Besides, followed by recurrent lesion in lung, the patient achieved a disease stabilization for 13 months [83]. Researchers further investigated whether the tumor rejection was attributed to ERBB2IP mutation specific CD4^+^ T cell. The patient with refractory tumor after treatment received a second adoptive T cell transfer which contained more than 95% ERBB2IP mutation specific CD4^+^ T cells [83]. Unexpectedly, the tumor was eliminated more quickly and potently than first T cell injection, and the tumor was observed obvious regression as early as following 1st month [83]. Apart from selection and expansion from TIL, neoantigen specific T cell could also be obtained from TCR-engineered T cell [84]. Commonly, neoantigen specific T cell is prepared by transferring genetic material which could encode corresponding TCR or synthetic chimeric antigen receptor [84–86].

One of determinants for efficacy of immune checkpoint inhibitor is pre-existing tumor specific T cell, so it is feasible to combine adoptive T cell transfer and immune checkpoint blockade especially for patient with severely inhibited immunity [5, 84]. It was noticed that combination of anti-immune checkpoint and T cell transfer targeting tumor associated antigen induced tumor eradication successfully [87]. Limited by complicated procedure for obtaining neoantigen-specific T cell, study verifying the effect of combination therapy of neoantigen-specific T cell transfer and ICI is unavailable. However, it has been verified that the effect of TIL transfer could be boosted by immune checkpoint blockade [88]. In theory, combination therapy of immune checkpoint inhibitor and neoantigen specific adoptive T cell transfer is a reasonable strategy in the absence of pre-existing tumor specific T cell, but the actual efficacy should be investigated further.

## Conclusion

Mutation is a double-edged sword for tumor growth. It contributes to tumorigenesis and progression, but in the meanwhile, the mutation could be recognized by host immunity and lead to tumor elimination. It has been confirmed that neoantigen specific T cell activity is the main determinant of immunotherapy. Thus, strategies targeting neoantigen receive intensive attention for the synthetic effect with other immunotherapies such as immune checkpoint inhibitor. Based on NGS and public databases, multiple algorithms were established and optimized for neoantigen prediction in silico, which further propelled development of neoantigen cancer vaccine and T cell transfer. In terms of the importance of pre-existing anti-tumor immune response for immune checkpoint inhibitor, we believe personalized neoantigen-based treatment would be a promising synthetic strategy.

## Abbreviations

Alg8: Asparagine-linked glycosylation 8
APC: antigen presentation cell
CTLA-4: cytotoxic T lymphocyte-associated antigen-4
ERBB2IP: erbb2 interacting protein
HLA: human leukocyte antigen
Lama4: Laminin alpha subunit 4
MHC: major histocompatibility complex
MMR: mismatch repair
MSI: microsatellite instability
NGS: next generation sequencing
NSCLC: non-small cell lung cancer
PD-1: programmed cell death protein 1
PD-L1: programmed cell death ligand 1
PFS: progression-free survival
TAP: transporter associated with antigen processing
TCR: T cell receptor
TGF-β: transforming growth factor-β
TIL: tumor infiltrating lymphocyte
Tim-3: T cell immunoglobulin and mucin domain-containing protein-3
TMB: tumor mutation burden
WT: wild type

## References

[1] Schumacher TN, Schreiber RD (2015). Neoantigens in cancer immunotherapy. Science.

[2] Yu S, Li A, Liu Q, Li T, Yuan X, Han X (2017). Chimeric antigen receptor T cells: a novel therapy for solid tumors. J Hematol Oncol..

[3] Yu S, Li A, Liu Q, Yuan X, Xu H, Jiao D (2017). Recent advances of bispecific antibodies in solid tumors. J Hematol Oncol..

[4] Hailemichael Y, Woods A, Fu T, He Q, Nielsen MC, Hasan F (2018). Cancer vaccine formulation dictates synergy with CTLA-4 and PD-L1 checkpoint blockade therapy. J Clin Invest..

[5] Yi M, Jiao D, Xu H, Liu Q, Zhao W, Han X (2018). Biomarkers for predicting efficacy of PD-1/PD-L1 inhibitors. Mol Cancer..

[6] Chen DS, Mellman I (2013). Oncology meets immunology: the cancer-immunity cycle. Immunity.

[7] Liu D, Wang S, Bindeman W (2017). Clinical applications of PD-L1 bioassays for cancer immunotherapy. J Hematol Oncol..

[8] Neefjes J, Jongsma ML, Paul P, Bakke O (2011). Towards a systems understanding of MHC class I and MHC class II antigen presentation. Nat Rev Immunol.

[9] Hellmann MD, Callahan MK, Awad MM, Calvo E, Ascierto PA, Atmaca A (2018). Tumor mutational burden and efficacy of nivolumab monotherapy and in combination with ipilimumab in small-cell lung cancer. Cancer Cell.

[10] Goodman AM, Kato S, Bazhenova L, Patel SP, Frampton GM, Miller V (2017). Tumor mutational burden as an independent predictor of response to immunotherapy in diverse cancers. Mol Cancer Ther.

[11] Rooney MS, Shukla SA, Wu CJ, Getz G, Hacohen N (2015). Molecular and genetic properties of tumors associated with local immune cytolytic activity. Cell.

[12] Gong J, Chehrazi-Raffle A, Reddi S, Salgia R (2018). Development of PD-1 and PD-L1 inhibitors as a form of cancer immunotherapy: a comprehensive review of registration trials and future considerations. J Immunother Cancer..

[13] Turajlic S, Litchfield K, Xu H, Rosenthal R, McGranahan N, Reading JL (2017). Insertion-and-deletion-derived tumour-specific neoantigens and the immunogenic phenotype: a pan-cancer analysis. Lancet Oncol..

[14] Rizvi NA, Hellmann MD, Snyder A, Kvistborg P, Makarov V, Havel JJ (2015). Cancer immunology. Mutational landscape determines sensitivity to PD-1 blockade in non-small cell lung cancer. Science.

[15] Le DT, Durham JN, Smith KN, Wang H, Bartlett BR, Aulakh LK (2017). Mismatch repair deficiency predicts response of solid tumors to PD-1 blockade. Science.

[16] Carbone DP, Reck M, Paz-Ares L, Creelan B, Horn L, Steins M (2017). First-line nivolumab in stage IV or recurrent non-small-cell lung cancer. N Engl J Med.

[17] Li GM (2008). Mechanisms and functions of DNA mismatch repair. Cell Res.

[18] Overman MJ, McDermott R, Leach JL, Lonardi S, Lenz HJ, Morse MA (2017). Nivolumab in patients with metastatic DNA mismatch repair-deficient or microsatellite instability-high colorectal cancer (CheckMate 142): an open-label, multicentre, phase 2 study. Lancet Oncol..

[19] Kim ST, Cristescu R, Bass AJ, Kim KM, Odegaard JI, Kim K (2018). Comprehensive molecular characterization of clinical responses to PD-1 inhibition in metastatic gastric cancer. Nat Med.

[20] Janjigian YY, Sanchez-Vega F, Jonsson P, Chatila WK, Hechtman JF, Ku GY (2018). Genetic predictors of response to systemic therapy in esophagogastric cancer. Cancer Discov.

[21] Anagnostou V, Smith KN, Forde PM, Niknafs N, Bhattacharya R, White J (2017). Evolution of neoantigen landscape during immune checkpoint blockade in non-small cell lung cancer. Cancer Discov.

[22] Long J, Lin J, Wang A, Wu L, Zheng Y, Yang X (2017). PD-1/PD-L blockade in gastrointestinal cancers: lessons learned and the road toward precision immunotherapy. J Hematol Oncol..

[23] Snyder A, Makarov V, Merghoub T, Yuan J, Zaretsky JM, Desrichard A (2014). Genetic basis for clinical response to CTLA-4 blockade in melanoma. N Engl J Med.

[24] Routy B, Le Chatelier E, Derosa L, Duong CPM, Alou MT, Daillere R (2018). Gut microbiome influences efficacy of PD-1-based immunotherapy against epithelial tumors. Science.

[25] Matson V, Fessler J, Bao R, Chongsuwat T, Zha Y, Alegre ML (2018). The commensal microbiome is associated with anti-PD-1 efficacy in metastatic melanoma patients. Science.

[26] Gopalakrishnan V, Spencer CN, Nezi L, Reuben A, Andrews MC, Karpinets TV (2018). Gut microbiome modulates response to anti-PD-1 immunotherapy in melanoma patients. Science.

[27] Yi M, Yu S, Qin S, Liu Q, Xu H, Zhao W (2018). Gut microbiome modulates efficacy of immune checkpoint inhibitors. J Hematol Oncol..

[28] Hackl H, Charoentong P, Finotello F, Trajanoski Z (2016). Computational genomics tools for dissecting tumour-immune cell interactions. Nat Rev Genet.

[29] Snyder A, Chan TA (2015). Immunogenic peptide discovery in cancer genomes. Curr Opin Genet Dev.

[30] Ding L, Wendl MC, McMichael JF, Raphael BJ (2014). Expanding the computational toolbox for mining cancer genomes. Nat Rev Genet.

[31] Pabinger S, Dander A, Fischer M, Snajder R, Sperk M, Efremova M (2014). A survey of tools for variant analysis of next-generation genome sequencing data. Brief Bioinform.

[32] Warren RL, Choe G, Freeman DJ, Castellarin M, Munro S, Moore R (2012). Derivation of HLA types from shotgun sequence datasets. Genome Med..

[33] Szolek A, Schubert B, Mohr C, Sturm M, Feldhahn M, Kohlbacher O (2014). OptiType: precision HLA typing from next-generation sequencing data. Bioinformatics.

[34] Nariai N, Kojima K, Saito S, Mimori T, Sato Y, Kawai Y (2015). HLA-VBSeq: accurate HLA typing at full resolution from whole-genome sequencing data. BMC Genomics..

[35] Huang Y, Yang J, Ying D, Zhang Y, Shotelersuk V, Hirankarn N (2015). HLAreporter: a tool for HLA typing from next generation sequencing data. Genome Med..

[36] Kim S, Kim HS, Kim E, Lee MG, Shin EC, Paik S (2018). Neopepsee: accurate genome-level prediction of neoantigens by harnessing sequence and amino acid immunogenicity information. Ann Oncol.

[37] Hoof I, Peters B, Sidney J, Pedersen LE, Sette A, Lund O (2009). NetMHCpan, a method for MHC class I binding prediction beyond humans. Immunogenetics.

[38] Stambrook PJ, Maher J, Farzaneh F (2017). Cancer immunotherapy: whence and whither. Mol Cancer Res.

[39] Rock KL, Reits E, Neefjes J (2016). Present yourself! By MHC class I and MHC class II molecules. Trends Immunol.

[40] Josephs TM, Grant EJ, Gras S (2017). Molecular challenges imposed by MHC-I restricted long epitopes on T cell immunity. Biol Chem.

[41] Karosiene E, Lundegaard C, Lund O, Nielsen M (2012). NetMHCcons: a consensus method for the major histocompatibility complex class I predictions. Immunogenetics.

[42] Andreatta M, Karosiene E, Rasmussen M, Stryhn A, Buus S, Nielsen M (2015). Accurate pan-specific prediction of peptide-MHC class II binding affinity with improved binding core identification. Immunogenetics.

[43] Bomberger JM, Ely KH, Bangia N, Ye S, Green KA, Green WR (2014). *Pseudomonas aeruginosa* Cif protein enhances the ubiquitination and proteasomal degradation of the transporter associated with antigen processing (TAP) and reduces major histocompatibility complex (MHC) class I antigen presentation. J Biol Chem.

[44] Nielsen M, Lundegaard C, Lund O, Kesmir C (2005). The role of the proteasome in generating cytotoxic T-cell epitopes: insights obtained from improved predictions of proteasomal cleavage. Immunogenetics.

[45] Zhang GL, Petrovsky N, Kwoh CK, August JT, Brusic V (2006). PRED(TAP): a system for prediction of peptide binding to the human transporter associated with antigen processing. Immunome Res..

[46] Trolle T, Nielsen M (2014). NetTepi: an integrated method for the prediction of T cell epitopes. Immunogenetics.

[47] Lee CH, Yelensky R, Jooss K, Chan TA (2018). Update on tumor neoantigens and their utility: why it is good to be different. Trends Immunol.

[48] Kaufman HL, Kohlhapp FJ, Zloza A (2015). Oncolytic viruses: a new class of immunotherapy drugs. Nat Rev Drug Discov..

[49] Marelli G, Howells A, Lemoine NR, Wang Y (2018). Oncolytic viral therapy and the immune system: a double-edged sword against cancer. Front Immunol..

[50] Parato KA, Breitbach CJ, Le Boeuf F, Wang J, Storbeck C, Ilkow C (2012). The oncolytic poxvirus JX-594 selectively replicates in and destroys cancer cells driven by genetic pathways commonly activated in cancers. Mol Ther.

[51] Yin J, Markert JM, Leavenworth JW (2017). Modulation of the intratumoral immune landscape by oncolytic herpes simplex virus virotherapy. Front Oncol..

[52] Woller N, Gurlevik E, Fleischmann-Mundt B, Schumacher A, Knocke S, Kloos AM (2015). Viral infection of tumors overcomes resistance to PD-1-immunotherapy by broadening neoantigenome-directed T-cell responses. Mol Ther.

[53] Chen CY, Hutzen B, Wedekind MF, Cripe TP (2018). Oncolytic virus and PD-1/PD-L1 blockade combination therapy. Oncolytic Virother..

[54] Bartee E, Li Z (2017). In vivo and in situ programming of tumor immunity by combining oncolytics and PD-1 immune checkpoint blockade. Exp Hematol Oncol..

[55] Tremblay-LeMay R, Rastgoo N, Chang H (2018). Modulating PD-L1 expression in multiple myeloma: an alternative strategy to target the PD-1/PD-L1 pathway. J Hematol Oncol..

[56] Zamarin D, Ricca JM, Sadekova S, Oseledchyk A, Yu Y, Blumenschein WM (2018). PD-L1 in tumor microenvironment mediates resistance to oncolytic immunotherapy. J Clin Invest..

[57] Demaria S, Golden EB, Formenti SC (2015). Role of local radiation therapy in cancer immunotherapy. JAMA Oncol..

[58] Alexander GS, Palmer JD, Tuluc M, Lin J, Dicker AP, Bar-Ad V (2016). Immune biomarkers of treatment failure for a patient on a phase I clinical trial of pembrolizumab plus radiotherapy. J Hematol Oncol..

[59] Formenti SC, Demaria S (2012). Radiation therapy to convert the tumor into an in situ vaccine. Int J Radiat Oncol Biol Phys.

[60] Esposito A, Criscitiello C, Curigliano G (2015). Immune checkpoint inhibitors with radiotherapy and locoregional treatment: synergism and potential clinical implications. Curr Opin Oncol.

[61] Ko EC, Formenti SC (2018). Radiotherapy and checkpoint inhibitors: a winning new combination?. Ther Adv Med Oncol..

[62] Liu Y, Dong Y, Kong L, Shi F, Zhu H, Yu J (2018). Abscopal effect of radiotherapy combined with immune checkpoint inhibitors. J Hematol Oncol..

[63] Aboudaram A, Modesto A, Chaltiel L, Gomez-Roca C, Boulinguez S, Sibaud V (2017). Concurrent radiotherapy for patients with metastatic melanoma and receiving anti-programmed-death 1 therapy: a safe and effective combination. Melanoma Res.

[64] Fiorica F, Belluomini L, Stefanelli A, Santini A, Urbini B, Giorgi C (2018). Immune checkpoint inhibitor nivolumab and radiotherapy in pretreated lung cancer patients: efficacy and safety of combination. Am J Clin Oncol.

[65] Jiang W, Chan CK, Weissman IL, Kim BYS, Hahn SM (2016). Immune priming of the tumor microenvironment by radiation. Trends Cancer..

[66] Marin-Acevedo JA, Soyano AE, Dholaria B, Knutson KL, Lou Y (2018). Cancer immunotherapy beyond immune checkpoint inhibitors. J Hematol Oncol..

[67] Aurisicchio L, Pallocca M, Ciliberto G, Palombo F (2018). The perfect personalized cancer therapy: cancer vaccines against neoantigens. J Exp Clin Cancer Res..

[68] Wang YQ, Wu J, Fan QZ, Zhou M, Yue ZG, Ma GH (2014). Novel vaccine delivery system induces robust humoral and cellular immune responses based on multiple mechanisms. Adv Healthc Mater..

[69] Li M, Li Y, Peng K, Wang Y, Gong T, Zhang Z (2017). Engineering intranasal mRNA vaccines to enhance lymph node trafficking and immune responses. Acta Biomater.

[70] Gubin MM, Zhang X, Schuster H, Caron E, Ward JP, Noguchi T (2014). Checkpoint blockade cancer immunotherapy targets tumour-specific mutant antigens. Nature.

[71] Ok CY, Young KH (2017). Checkpoint inhibitors in hematological malignancies. J Hematol Oncol..

[72] Munn DH, Bronte V (2016). Immune suppressive mechanisms in the tumor microenvironment. Curr Opin Immunol.

[73] Sahin U, Derhovanessian E, Miller M, Kloke BP, Simon P, Lower M (2017). Personalized RNA mutanome vaccines mobilize poly-specific therapeutic immunity against cancer. Nature.

[74] Ott PA, Hu Z, Keskin DB, Shukla SA, Sun J, Bozym DJ (2017). An immunogenic personal neoantigen vaccine for patients with melanoma. Nature.

[75] Furness AJ, Quezada SA, Peggs KS (2016). Neoantigen heterogeneity: a key driver of immune response and sensitivity to immune checkpoint blockade?. Immunotherapy..

[76] McGranahan N, Furness AJ, Rosenthal R, Ramskov S, Lyngaa R, Saini SK (2016). Clonal neoantigens elicit T cell immunoreactivity and sensitivity to immune checkpoint blockade. Science.

[77] Mittal D, Gubin MM, Schreiber RD, Smyth MJ (2014). New insights into cancer immunoediting and its three component phases-elimination, equilibrium and escape. Curr Opin Immunol.

[78] DuPage M, Mazumdar C, Schmidt LM, Cheung AF, Jacks T (2012). Expression of tumour-specific antigens underlies cancer immunoediting. Nature.

[79] Carreno BM, Magrini V, Becker-Hapak M, Kaabinejadian S, Hundal J, Petti AA (2015). Cancer immunotherapy. A dendritic cell vaccine increases the breadth and diversity of melanoma neoantigen-specific T cells. Science.

[80] Marshall HT, Djamgoz MBA (2018). Immuno-oncology: emerging targets and combination therapies. Front Oncol..

[81] Kato T, Matsuda T, Ikeda Y, Park JH, Leisegang M, Yoshimura S (2018). Effective screening of T cells recognizing neoantigens and construction of T-cell receptor-engineered T cells. Oncotarget..

[82] Medavaram S, Zhang Y (2018). Emerging therapies in advanced hepatocellular carcinoma. Exp Hematol Oncol..

[83] Tran E, Turcotte S, Gros A, Robbins PF, Lu YC, Dudley ME (2014). Cancer immunotherapy based on mutation-specific CD4+ T cells in a patient with epithelial cancer. Science.

[84] Matsuda T, Leisegang M, Park JH, Ren L, Kato T, Ikeda Y (2018). Induction of neoantigen-specific cytotoxic T Cells and construction of T-cell receptor-engineered T cells for ovarian cancer. Clin Cancer Res.

[85] Met O, Jensen KM, Chamberlain CA, Donia M, Svane IM (2018). Principles of adoptive T cell therapy in cancer. Semin Immunopathol.

[86] Rataj F, Kraus FBT, Chaloupka M, Grassmann S, Heise C, Cadilha BL (2018). PD1-CD28 fusion protein enables CD4+ T Cell help for adoptive T cell therapy in models of pancreatic cancer and non-hodgkin lymphoma. Front Immunol..

[87] Chong EA, Melenhorst JJ, Lacey SF, Ambrose DE, Gonzalez V, Levine BL (2017). PD-1 blockade modulates chimeric antigen receptor (CAR)-modified T cells: refueling the CAR. Blood.

[88] Ligtenberg MA, Pico de Coana Y, Shmushkovich T, Yoshimoto Y, Truxova I, Yang Y (2018). Self-delivering RNAi targeting PD-1 improves tumor-specific T cell functionality for adoptive cell therapy of malignant melanoma. Mol Ther.

[89] Lundegaard C, Lamberth K, Harndahl M, Buus S, Lund O, Nielsen M (2008). NetMHC- 3.0: accurate web accessible predictions of human, mouse and monkey MHC class I affinities for peptides of length 8–11. Nucleic Acids Res.

[90] Nielsen M, Andreatta M (2016). NetMHCpan-3.0; improved prediction of binding to MHC class I molecules integrating information from multiple receptor and peptide length datasets. Genome Med..

[91] Jorgensen KW, Rasmussen M, Buus S, Nielsen M (2014). NetMHCstab—predicting stability of peptide-MHC-I complexes; impacts for cytotoxic T lymphocyte epitope discovery. Immunology.

[92] Zhang H, Lund O, Nielsen M (2009). The PickPocket method for predicting binding specificities for receptors based on receptor pocket similarities: application to MHC-peptide binding. Bioinformatics.

[93] Schubert B, Walzer M, Brachvogel HP, Szolek A, Mohr C, Kohlbacher O (2016). FRED 2: an immunoinformatics framework for Python. Bioinformatics.

[94] Larsen MV, Lundegaard C, Lamberth K, Buus S, Lund O, Nielsen M (2007). Large-scale validation of methods for cytotoxic T-lymphocyte epitope prediction. BMC Bioinform.

[95] Stranzl T, Larsen MV, Lundegaard C, Nielsen M (2010). NetCTLpan: pan-specific MHC class I pathway epitope predictions. Immunogenetics.

[96] Hundal J, Carreno BM, Petti AA, Linette GP, Griffith OL, Mardis ER (2016). pVAC-Seq: a genome-guided in silico approach to identifying tumor neoantigens. Genome Med..

[97] Schubert B, Brachvogel HP, Jurges C, Kohlbacher O (2015). EpiToolKit—a web-based workbench for vaccine design. Bioinformatics.

[98] Donnes P, Kohlbacher O (2005). Integrated modeling of the major events in the MHC class I antigen processing pathway. Protein Sci.

